# Bacterial skin infection caused by a plant pathogen Kosakonia cowanii: identification with the MALDI Biotyper sirius one and susceptibility testing

**DOI:** 10.1099/acmi.0.000923.v3

**Published:** 2025-01-28

**Authors:** John Merlino, Kiora Pillay, Sophia Rizzo, Sai Rupa Baskar, Daniel Seed, Steven Siarakas, Ravin Hettiarachchi, Genevieve McKew, Timothy Gray

**Affiliations:** 1 Department of Microbiology and Infectious Diseases, NSW Health Pathology, Concord Hospital, Sydney, Australia; 2 Department of Infectious Disease and Immunology, School of Medical Sciences, Faculty of Medicine and Health, University of Sydney, Sydney, Australia; 3 Faculty of Medicine and Health, Concord Clinical School, University of Sydney, Sydney, Australia

**Keywords:** cephalosporins, European Committee on Antimicrobial Susceptibility Testing (EUCAST) guidelines, *Kosakonia cowanii*, MALDI-TOF biotyper sirius one, opportunistic human pathogen, *Pantoea *spp., skin infections

## Abstract

*Kosakonia cowanii* is a Gram-negative bacillus belonging to the order Enterobacterales. *K. cowanii* is a plant pathogen; we report the isolation of this organism from a gardening puncture injury from a plant. Pus examined from the site of infection showed the infecting organism as *K. cowanii* by MALDI-TOF MS Biotyper Sirius One and 16S rRNA analysis. The organism failed to be identified biochemically by the Vitek 2 XL identification system as *Kosakonia* spp. The Gram-negative bacilli on the system were biochemically identified as *Pantoea* spp. Difficulties in species identification biochemically suggest that *K. cowanii* might potentially represent an underestimated opportunistic human pathogen in skin infections associated with plant injuries. The MALDI-TOF MS Biotyper Sirius One software was found to be a fast and reliable identification of the organism. The isolate was found to be susceptible to first-generation cephalosporins, both cefalexin and cefazolin, by disc diffusion and Vitek 2 XL, even though antibiotic breakpoints currently do not exist using European Committee on Antimicrobial Susceptibility Testing or Clinical and Laboratory Standards Institute (CLSI) guidelines for this organism.

## Data Summary

The authors confirm that all data associated with this work are reported within this article.

## Introduction

*Kosakonia cowanii*, previously identified as *Enterobacter cowanii*, was discovered as a novel species of the Enterobacteriaceae family in 2000 [1]. Until 2013, it remained classified as an *Enterobacter* spp. until differentiated by phylogeny following MLSA prompting proposed reclassification [2]. *K. cowanii* has largely been considered an environmental isolate, existing as both a plant pathogen and as part of some plant phylloplanes [3]. Reports of *K. cowanii* pathogenicity in humans are much less common, with current literature describing rare cases of rhabdomyolysis due to bacteraemia and acute cholecystitis due to the organism [4,5]. This report describes the isolation of *K. cowanii* from pus from a skin wound infection.

### Case presentation

A female in her 90's was referred to the emergency department for removal of a foreign body following a gardening puncture injury from a plant sustained 2 weeks prior to presentation. She initially presented to her general practitioner with left palm erythema and swelling. She received two courses of oral cefalexin which she had completed prior to presentation. She was systemically well with no fevers. On presentation, she was afebrile, and haemodynamically stable, and a local hand exam demonstrated a non-tender swelling over her left thenar eminence, with no signs of spreading erythema. An ultrasound demonstrated the presence of a retained foreign body. She was discussed with the plastic surgical team and returned electively for removal of the foreign body. She underwent an elliptical excision over the abscess, removal of the foreign body and irrigation with hydrogen peroxide and normal saline. Expressed pus was sent for microbiology culture and susceptibility testing. She was given an empirical course of oral cefalexin 500 mg QID for 5 days. Given symptom resolution, no change to her antibiotic therapy was made.

## Laboratory investigations

### Methods and results

Pus was received in the laboratory for microbiology culture and susceptibility.

### Microscopy

**Fig. 1.**
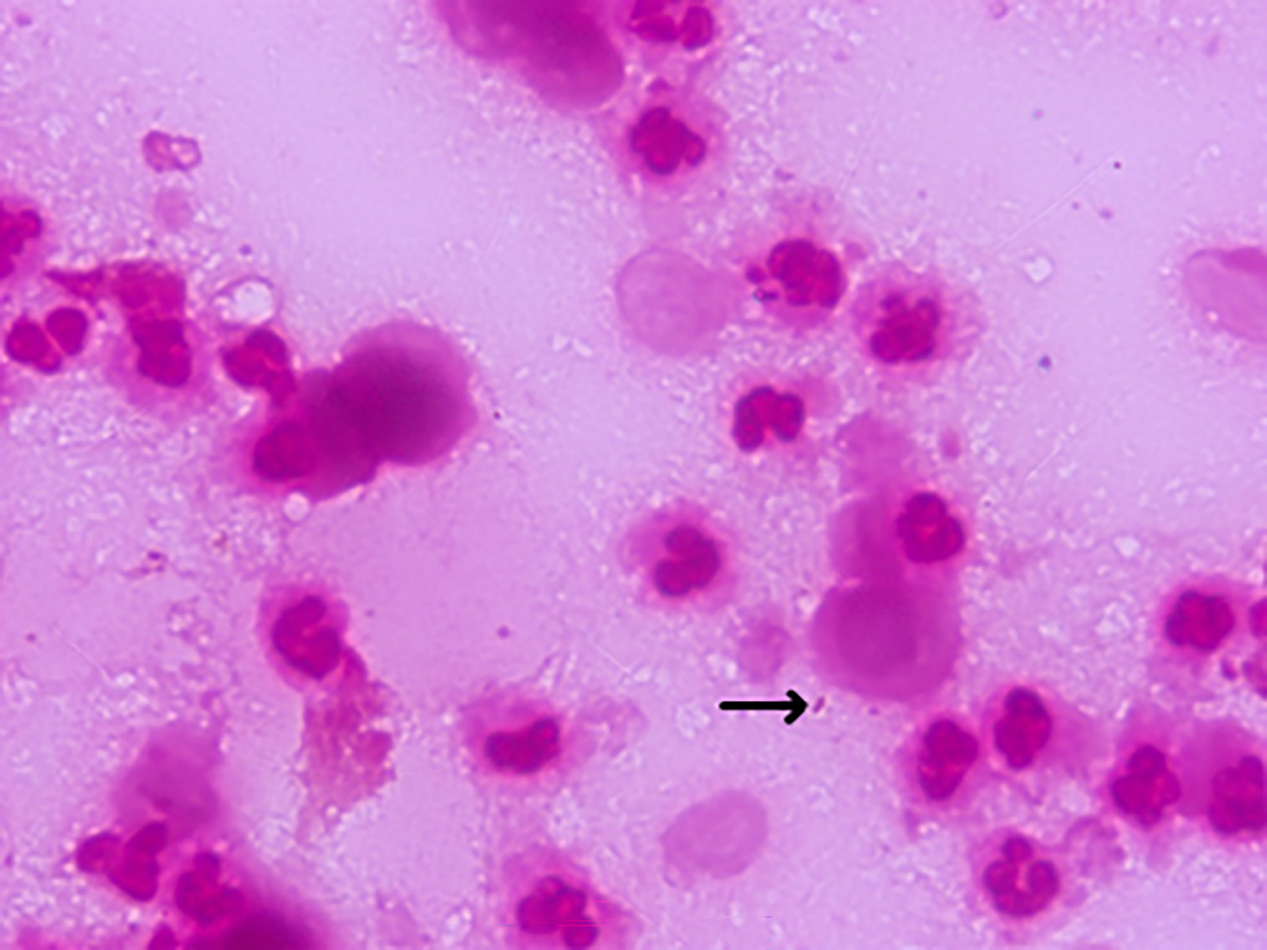
Gram stain of pus showing scant Gram-negative bacilli among polymorphonuclear cells.

Gram stain of the pus showed numerous polymorphonuclear cells with scanty Gram-negative bacilli (Fig. 1).

### Bacterial culture

Cultures grew a Gram-negative bacillus on Columbia horse blood agar (Thermo Fisher, Australia) aerobically at 37 °C in 5% CO_2_ after 24 h incubation and anaerobically after 48 h incubation. On MacConkey agar at 37 °C in ambient air, colonies appeared as weak lactose fermenters with a clear yellow to pink pigment. On Brilliance chromogenic agar (Thermo Fisher, Australia), the organism grew blue in colour.

### Bacterial identification

The Gram-negative bacillus was identified as *K. cowanii* using the MALDI-TOF MS Biotyper Sirius One (Bruker, Germany). The organism gave an average score of 2.3 for *K. cowanii* from five consecutive readings and ranged from 2.20 to 2.41. No formic acid was used prior to running the isolate through the MALDI-TOF MS. The Bruker identification database used was MBT IVD Library revision J (2022). Biochemical identification of the organism was performed on the Vitek 2 XL (bioMérieux, Australia) using the Vitek GN card (Card 21341) as described by the manufacturer. The Gram-negative bacilli on the system were biochemically identified as *Pantoea* spp. The isolate was repeated on the Vitek 2 XL system for confirmation with identical results with 98% probability as *Pantoea* spp. with a Bio score: 0607730551460010.

### 16S rRNA analysis

Species identification was confirmed by partial sequencing of the 16S rRNA gene as described previously [6]. The Gram-negative bacilli showed 100% identity using the National Center for Biotechnology Information blast search to the 16S rRNA of the *K. cowanii* type strain.

### Antibiotic susceptibility

Susceptibility testing was performed by disc diffusion and the Vitek 2 XL system and interpreted using European Committee on Antimicrobial Susceptibility Testing (EUCAST) (version 13.1) guidelines [7]. Susceptibility results are shown in Table 1.

**Table 1.** Antibiotic susceptibility results for the *Kosokonia cowanii* clinical isolate

Antibiotic substance	Zone diameter (mm)*	MIC (mg l^−1^) on Vitek 2 XL†	Interpretation
Ampicillin	≤6	≥32	R
Amoxicillin/clavulanic acid	21	≤4	S
Cefoxitin	21	≤4	S
Cefalexin	21	nt	na
Cefazolin	23	≤4	na
Ceftriaxone	28	≤0.25	S
Cefepime	31	≤0.12	S
Piperacillin/tazobactam	21	≤4	S
Gentamicin	17	≤1	S
Amikacin	20	≤1	S
Tobramycin	19	≤1	S
Ertapenem	nt	≤12	S
Meropenem	24	≤0.25	S
Ciprofloxacin	28	≤0.06	S
Trimethoprim/sulfamethoxazole	22	≤20	S

na, not available, currently no EUCAST breakpoints exist for systemic infections for cefazolin and cephalexin; breakpoints only for uncomplicated urines.

* Zone diameters as determined by discs using EUCAST guidelines for *K. cowanii* clinical isolate.

† Interpretation according to EUCAST criteria for Enterobacterales (breakpoint tables for interpretation of MICs and zone diameters, Version 13.1, 2018; https://www.eucast.org/.

nt, not tested; this antibiotic was not tested on this panel.

## Discussion and conclusions

In 2013, *K. cowanii*, a member of the Enterobacterales family, was excluded from the *Enterobacter* genus and included in five new genera: *Lelliottia*, *Pluralibacter*, *Kosakonia*, *Cronobacter* and *Enterobacter*. *Kosakonia* spp. (*Kosakonia radicincitans*, *Kosakonia sacchari*, *Kosakonia oryzae*, *K. cowanii* and *Kosakonia arachidis*) are usually regarded as plant pathogens [5,8]. Very little epidemiological data apart from a few clinical cases exist in the literature on the occurrence of *Kosakonia* spp. in human infections. Such organisms are seldom included in human biochemical diagnostic reaction databases. As seen in this case, the Vitek 2 XL biochemical database reports the organism *K. cowanii* as *Pantoea* spp. with 98% probability. Biochemically, Procop *et al.* suggest that even though both *K. cowanii* and *Pantoea* spp. are negative for lysine, ornithine decarboxylases and arginine dihydrolase, tests useful for differentiation are both negative for malonate utilization and fermentation of dulcitol and sorbitol which are both negative for *Pantoea* spp. [9]. Difficulties in species identification biochemically suggest that potentially, *K. cowanii* might represent an underestimated human pathogen in skin infections associated with plant injuries. The patient’s clinical history does not help in differentiating the two organisms since both *Pantoea* spp. and *K. cowanii* are opportunistic pathogens in immunocompromised patients with associated infections acquired from vegetation penetrating the skin. New diagnostic tools such as the MALDI-TOF MS Biotyper Sirius One with an updated database with scores greater than 2 provide a fast reliable identification of the infecting organism with further 16 s rRNA analysis if confirmation is required as described in this manuscript.

Antibiotic susceptibility testing of *K. cowanii* was performed using EUCAST guidelines both by disc diffusion and Vitek 2 XL, and results and susceptibility interpretations are shown in Table 1. Although EUCAST or CLSI has not published first-generation cephalosporin breakpoints (disc diameters or minimum inhibitory concentration) for non-urinary Enterobacterales infections, the isolate tested susceptible to cefalexin and cefazolin using ‘uncomplicated urinary tract infection’ and ‘infections originating from the urinary tract’ breakpoints, respectively, by both disc diffusion and Vitek 2 XL. *K. cowanii* does not appear on the EUCAST ‘Expected Resistant Phenotype’ version 1.2 January 2023 [10]. Our antibiotic susceptibility profile as shown in Table 1 showed that the isolate was *ampC* gene negative, and our results confirm Bhatti *et al.*’s sequence findings that *Kosakonia cowanii* sequenced to date lack the *ampC* gene [11,12].

## Abbreviations

CLSI: Clinical and Laboratory Standards Institute
EUCAST: European Committee on Antimicrobial Susceptibility Testing
QID: four times a day
